# The Traumatic Neurosis and Intelligent Psychotherapy

**Published:** 1911-05

**Authors:** Tom A. Williams

**Affiliations:** Washington, D. C.; Foreign Corresp. Memb. Soc. de Neurologie; Soc. de Physchologic, Paris; Assoc. Memb. Clin. Soc. de Mental Med.


### THE TRAUMATIC NEUROSIS AND INTELLIGENT PSYCHOTHERAPY.

By Tom A. Williams, M.D., Washington, D. C.

*Foreign Corresp. Memb. Soc. de Neurologie; Soc. de Physchologic, Paris; Assoc. Memb. Clin. Soc. de Mental Med.;*

The pessimism, due to the want of success which has hitherto characterized medical efforts against the hystero-neurasthenic syndrome induced by industrial accidents, and especially those on railways, bids fair to be replaced by a very different attitude, thanks to the illumination of the whole subject of hysteria which we owe to the insight and energy of Babinski (1), who has effectually shown the purely fantastic nature of the hysteria described in the text books after the traditions of Charcot.

It is unfortunate that some clinicians, and more especially some neurologists, have not taken the trouble to study the mass of evidence about hysteria which has been accumulated since Charcot's day, and continue to write in some such strain as the following,—thus Church and Peters in 1908, editors, quote only the older article of Dutil,* saying, "a certain number of elementary phenomena, sensations and images are not preserved and appear to be repressed in the realm of consciousness." In addition, there are a number of organic phenomena—disturbances of nutrition, trophic and vaso-motor disorders of a neurotic charter." "The stigmata tend to persist as long as the affection lasts." "In the great majority of hysterias the visual field is found concentrically contracted." "The red visual field exceeds the blue." "Both anaesthesia and hyperaesthesia, etc., are not very rare." "The trophic accidents of hysteria are of recent recognition" (an extraordinary statement). "Even cutaneous gangrene has been recorded." "Neurotic oedema usually appears in parts hysterically affected." "Muscular atrophies have been observed by a number of reliable observers."

Worse still write Saville in a recent lecture published in the Lancet: "I cannot agree that hysterics are invariably or especially susceptible to auto and hetero suggestions, or are more hypnotisable than non-hysterical subjects." But he gives no facts or reason for this belief, merely stating further that "alternation and tendency to change and evanescence of mental state, called caprice by Sydenham, is the peculiarity par excellence by which we recognize a hysterical mind. When this means of identification fails he seeks it in emotional instability, a tendency to abstraction or automatism, especially coupled with certain physical symptoms, as flushing and fainting. He naively rejects the observations of both Freud and Janet on the ground that "their studies are carried on in psychological clinics, where patients apply principally or solely on account of mental defects," and believes they take "too narrow a view, because they do not mention or explain circulatory and somatic symptoms, and also state that they occur

See note in La Traite de Medicine de Bonchard-Briscaud, Paris, 1905.

much less frequently to observers engaged in general medicine." Saville can know very little of the Saltpetriere clinic in making such a remark, for there the number of persons suffering from physical defects of the nervous system is enormous (2), and it is there that Freud first formulated his theory, and Janet still works. They do not mention the physical symptoms in the same naive way as formerly, because they have long ceased to regard certain of them as belonging to hysteria. Other neuroses (in the true sense), having now been delimited, and other conditions, such as palpitations, flushings, syncope and convulsions being capable of easy production, either direct by suggestion or indirectly via an emotion. Everyone surely must be familiar with the pallor, cold flushes and syncope of fear and the blush of shame as well as the convulsive attack of the tantrums of childhood. To invoke a hypothetical splanchno-neurosis to explain such everyday phenomena shows great ignorance of well-known data concerning the emotions.

The illustration he uses to enforce a long settled thesis that some hysterical phenomena at least are psychogenetic, is unfortunate at least, for the example he gives is that of antics, full description of which Meige and Freindell (3), long ago showed to be quite different from hysteria, and as a member of the national hospital staff has (3), translated the book, the knowledge it conveys should be common property.

As gratuitious and discredited for lack of evidence is his hypothesis that hysterical symptoms must be explained by focal modifications, by cerebral anemia due to vaso-motor instability. Science is very far indeed from such a hypothesis, and any premature adherence thereto, on the part of clinicians, only hinders the advance of the real knowledge gained by a study of the phenomena, by making them unconsciously distort the facts to conform to an explanation which appears scientific because physiological. The knowledge of neurological diagnosis needed to exclude definite organic perturbations of the nervous system, and an acquaintance with the modern work of Charcot's followers, indicates that here is only one fact certain and indisputable about the reactions of hystericals, and that is that they are "susceptible or production by suggestion and of removal by suggestion—persuasion." (4.)

This is not the occasion to refute such notions, nor even to indicate in extenso the reasons for dissent, for the writer's recent articles (5), have shown forth the evidence, and in a communication to the Congress on industrial accidents at Rome (6), the application of the doctrine that the primitive symptoms of traumatic neuroses are hysterical neither more or less, in that they are each and all "susceptible of production by suggestion and of removal by suggestion-persuasion." I say, primitive advisedly, because, although hysterical contracture, for example, is produced by suggestion, yet the shortening of the tendons which may follow its prolongation is the result of organic changes due to persistence of a faulty attitude, and cannot be removed by suggestion-persuasion. Again, although the anorexia of certain gastric neuroses is caused through suggestion and removable thereby, yet the emaciation, asthenia and gastric insufficiency which result, cannot be thus removed, for they are organic consequences secondary to starvation induced by the fixed idea, the erroneous belief, that food will not agree. (8).

Similarly, in the case which follows the loss of appetite, insomnia, emaciation and unhealthy tint of the skin were secondary to the mental worry concerning the circumstances in which he was placed, and through his fixed idea, the false belief that he was irretrievably damaged in his spinal cord, and would be unable to earn a living for himself and family, and his whole affective tone thus became morbid secondarily to an idea derived by suggestion, as will appear.

It is the familiar case of an incapacitated railway employee to whom I was called to determine whether or not there was organic disease of the nervous system. The fact that there was not is shown elsewhere in the full report of the case. The psycogenesis of the man's condition was evident in his fixed idea, due to the common belief of railroad employees that serious nervous disease may slowly ensue upon an accident. This common belief was strengthened by the injudicious sympathy and inquiries of his friends, and the doubtful prognosis of some medical men he had consulted. He "answered a thousand questions a day," he "did not know what to think about his health," and worries about his circumstances, he was "too much preoccupied with his health even to miss his wife," he had lost weight and appetite, had a sore throat, and wept much, and finally his attitude was strengthened by the lawyers who sought redress for him. He was cured within a month as a result of one interview, during which he was instructed in the role of ideas over bodily activity and the effects of worry and anxiety upon nutrition. In the certificate it was stated, "there is and has been no disease of the spinal cord or peripheral nerves at play in the induction of any of the symptoms which I find. The erroneous belief that there has been such an injury powerfully contributes to the anxiety which maintains his present state."

This anxiety is really not primitive, but is "induced" or "conditioned" by an idea, viz.: his belief in his injury. The whole forms what is known in psychopathology as a " " i. e., a system of ideas and feeling associated together. A morbid, complex idea which dominates a person to his detriment. The traumatic neurosis is the prototype of such a morbid complex, and when this is generally and clearly realized by his physicians, the disease will be so easily cured that it will cease to exist. Of course, I exclude simulation in this prognosis. A deliberate feiguer present a purely medicological problem.

It must not be forgotten that, owing to its powerful feeling-tone, a complex may remain or recur until that feeling-tone is saisfied dynamically, or replaced by another. A pecuniary solution may furnish the dynamic satisfaction of the feeling-tone of injuries in traumatic neuroses.

This, however, is not the only means of doing so, for even the indemnity acts only in virtue of the effect upon the patient's aumour propre, which may be flattered in many other ways, as by sympathy, understanding, etc.

The subject need not be conscious of these idea systems, for they may be merely intellectual potencies implicit in the psychic constitution derived from his environment. In the gross, these are most clearly manifest in what our attitude of detachment easily enables us to label the superstitions of alien peoples. Thus, the sufferings induced by the "gnawing fox" of the Japanese are made possible only by a deeply rooted belief in its existence. For example, a woman after labor declared she felt the "fox coming," this was her interpretation of the after-pains she felt. The great parade by the neighbors in attempting to prevent the fox's attack only reinforced the patient's apprehensions and soon a horrible convulsion signalized her seizure by the fox. Terror and convulsion held her until the exorciser was called. He declared that the fox would leave her at four o'clock the next day, provided certain offerings were placed on a certain tomb for it to eat. This simple suggestion caused her to dismiss her terror suddenly at the hour designated.

The crudeness of the mechanism in the case of this ignorant peasant need not make us smile, for our western case is very little better, as our illustration shows.

It is clear that the return of this man's functional capacity was the result of the enlightenment and skilful persuasion he received during our interview, seconded by his physician, who saw that immediate action followed an intellectual conviction which might not have been maintained against the counter-suggestions he would have received in the environment of invalidism which had grown up around him. It must be remembered that patients with a fixed idea become aboulic where other matters are concerned. Thus Brissaud 15), remarked of a patient who went into a fit when they gently attempted to extend the contracture of a limb which had lasted five years since the railway accident: "This contracture is his life."

Mysoneism, (16), the impossibility of adaption to unusual conditions, is common enough, and its intensity is proportional to the length of time during which the mental habit has persisted, as well as to the affection, so to speak, with which one's habit or defect has been cherished and the age at which they have been acquired, and in such persons conviction soon becomes inert if allowed to sleep.

The effects of an emotion, such as fear, quickly pass away unless they are maintained artificially—ideationally, as by suggestions, which need not necessarily be made after the event, but may be latent, as in the following case:

A girl was brought to Babinski (17), having become monoplegic upon receiving an electric shock while crossing a tramway line. This seemed liked paralysis not caused by suggestion, but after the symptom had been removed by persuasion, further inquiry elicited the fact that the patient had overheard some months previously a conversation between some electricians, who were speaking of the dangers arising from electric shocks of the above description. It is evident that upon experiencing the shock, there had flashed into the patient's mind a datum learned from the conversation she had overheard and apparently forgotten, and that this memory furnished the suggestion at the base of the palsy she developed.

A suggestion need not even be explicit, but it is often implicit in the whole conduct of those who surround us. It may occur in consequence of manifestly insincere attempts to minimize what the patient sees that from their very manner those who surround him believe to be gravely dangerous. Alarm is difficult to conceal, and is very contagious, but is soon dissipated when a reassuring idea is successfully implanted.

Some theorists believe that the trauma of fright increases susceptibly to "neurosis," by creating new physiologic dispositions in the spinal and lower neurones, but we have no evidence to show that such dispositions are modifiable volitionally. The true neuroses, that is functional affections of the lower nerve paths, the physical basis of which is at present unknown to us, can be neither acquired nor removed by psychic means. To telencephalic anomalies the word psychosis (18) should be applied, and the traumatic neurosis is of this type, for to explain the reaction of these patients we must invoke the labill differentiability of neopalial, psychic adaptation rather than the inherent neuric reflexes phylogenetically organized, which we call instincts.

Hence, traumatic neurosis is only one form of suggestion psychosis, for suggestion is sufficient and efficient, and no other alleged cause is even essential, that is to say, the condition is pure hysteria, for its primary symptoms are each "susceptible of production by ugestion and removal by suggestion-persuasion." (19.).

